# Water Proton Spin Relaxivities and Absolute Fluorescent Quantum Yields of Triply and Quadruply Mixed Lanthanide Oxide Nanoparticles

**DOI:** 10.3390/ijms27020959

**Published:** 2026-01-18

**Authors:** Abdullah Khamis Ali Al Saidi, Tirusew Tegafaw, Dejun Zhao, Ying Liu, Endale Mulugeta, Xiaoran Chen, Ziyi Lin, Hansol Lee, Ahrum Baek, Jihyun Kim, Yongmin Chang, Gang Ho Lee

**Affiliations:** 1 Department of Chemistry, College of Natural Sciences, Kyungpook National University, Taegu 41566, Republic of Korea; abdullahalsaidi0429@gmail.com (A.K.A.A.S.); tirukorea@gmail.com (T.T.); djzhao.chem@gmail.com (D.Z.); ly1124161@gmail.com (Y.L.); endexindex05@gmail.com (E.M.); tsukiyovo@gmail.com (X.C.); 01linziyi@gmail.com (Z.L.); 2 Department of Medical & Biological Engineering, Kyungpook National University, Taegu 41944, Republic of Korea; leehs9836@naver.com; 3 Institute of Biomedical Engineering Research, School of Medicine, Kyungpook National University, Taegu 41944, Republic of Korea; baxun@naver.com; 4 Department of Chemistry Education, Teachers’ College, Kyungpook National University, Taegu 41566, Republic of Korea; jkim23@knu.ac.kr; 5 Department of Molecular Medicine, School of Medicine, Kyungpook National University, Taegu 41944, Republic of Korea

**Keywords:** multicomponent mixed lanthanide oxide nanoparticle, water proton spin relaxivity, absolute quantum yield, fluorescence life time, multimodal imaging agent

## Abstract

Multicomponent mixed lanthanide oxide (MMLO) nanoparticles possess considerable potential as multimodal imaging agents because they integrate diverse excellent optical and magnetic properties within a single nanoparticle. Herein, we present triply and quadruply mixed lanthanide oxide nanoparticles, namely, gadolinium (Gd)/dysprosium (Dy)/europium (Eu) oxide (GDEO), Gd/Dy/terbium (Tb) oxide (GDTO), and Gd/Dy/Eu/Tb oxide (GDETO) nanoparticles. Gd^3+^ can strongly induce positive (T_1_) contrast in magnetic resonance imaging (MRI), Dy^3+^ and Tb^3+^ can generate negative (T_2_) contrast in MRI, and Eu^3+^ and Tb^3+^ emit visible photons that are applicable to fluorescence imaging (FI). All the nanoparticles were grafted with hydrophilic, biocompatible polyacrylic acid (PAA) to enhance colloidal stability and biocompatibility and further grafted with small amounts of an organic photosensitizer, 2,6-pyridinedicarboxylic acid (PDA), to obtain a high absolute fluorescent quantum yield (QY) with an extended fluorescent lifetime (τ). All PAA-MMLO and PAA/PDA-MMLO nanoparticles exhibited nearly monodispersed particle-size distributions with average particle diameters of ~2 nm and displayed considerably higher longitudinal (r_1_) and transverse (r_2_) water proton spin relaxivities than commercial molecular MRI contrast agents. The PAA/PDA-GDEO, PAA/PDA-GDTO, and PAA/PDA-GDETO nanoparticles exhibited high absolute QYs of 45, 29, and 61%, respectively, and long τ values of 1–2 ms, making them suitable for time-delayed noise-free fluorescence signal detection. These findings confirm the high potential of PAA-MMLO nanoparticles as T_1_ and/or T_2_ MRI contrast agents and PAA/PDA-MMLO nanoparticles as both T_1_ and/or T_2_ MRI and FI agents.

## 1. Introduction

Nanoparticles are ideal systems to incorporate different types of metal ions within the same system, allowing for property tunability and multifunctionality. This will enable them to have a wide range of applications, including catalysis [1], energy storage [2], lighting [3], food [4], biotechnology [5], drug delivery [6], and nanomedicine [7]. Lanthanides (Ln) have garnered significant attention owing to their unique magnetic, optical, and electrical properties, which stem from their distinctive 4f-electron configurations [8,9,10]. Specifically, lanthanide oxide nanoparticles can be used as imaging agents in magnetic resonance imaging (MRI) and fluorescent imaging (FI) because they can produce higher contrasts [11] and stronger fluorescent intensities [12,13,14] than their molecular counterparts. Therefore, multicomponent mixed lanthanide oxide (MMLO) nanoparticles will be valuable for advanced and expanded imaging applications, offering innovative solutions for both diagnosis and therapy [15,16].

As presented in Table 1, gadolinium (Gd^3+^) ion, with an ^8^S_7/2_ electron configuration, can efficiently reduce the longitudinal (T_1_) relaxation time of surrounding water proton spins [17,18,19], resulting in brighter (or positive) MR images. Conversely, dysprosium (Dy^3+^) and terbium (Tb^3+^) ions, which are characterized by ^6^H_15/2_ and^7^F_6_ electron configurations, respectively, can serve as negative (T_2_) MRI contrast agents by influencing the magnetic field in their vicinity, thereby reducing the transverse (T_2_) relaxation time of surrounding water proton spins [20,21,22,23,24]. This effect induces negative contrast, which darkens the image. Meanwhile, europium (Eu^3+^) ion, with its ^7^F_0_ electronic configuration [12,25,26], and Tb^3+^ [27,28,29] can function as FI agents, emitting red and green light upon excitation, respectively, making them invaluable for imaging biological systems. They exhibit exceptional photostability, prolonged fluorescence lifetimes, and atomic-like sharp emissions, rendering them useful for high-performance in vitro and in vivo FI applications such as cell labeling and sensing, optical coding of biomolecules, cancer targeting, drug delivery, and disease diagnosis [12,13,14].

A remarkable feature of lanthanide oxide nanoparticles is their ability to easily accommodate multiple Ln^3+^ within a single nanoparticle owing to their similar atomic and ionic radii and identical valence state (i.e., +3). For example, D-glucuronic acid-coated mixed gadolinium–dysprosium oxide nanoparticles have demonstrated effective performance as dual-modal positive T_1_ and negative T_2_ MRI contrast agents [30]. Tb/Eu and Dy/erbium (Er) oxide nanoparticles have also been evaluated for their photoluminescence (PL) efficiency, revealing many intense and sharply defined emission peaks, thereby expanding their applicability as FI agents [31].

Multicomponent mixed lanthanide oxide (MMLO) nanoparticles are more useful for biomedical applications than conventional doubly mixed lanthanide oxide nanoparticles. The integration of multiple Ln^3+^ offers MMLO nanoparticles significant potential for advancing medical imaging and diagnostic endeavors in monitoring treatment responses and guiding the targeted delivery of therapeutic payloads, which would ultimately advance precision medicine and therapeutic interventions.

In this study, we synthesized MMLO nanoparticles, namely, Gd/Dy/Eu oxide (GDEO), Gd/Dy/Tb oxide (GDTO), and Gd/Dy/Eu/Tb oxide (GDETO) nanoparticles. To enhance the colloidal stability and biocompatibility, the MMLO nanoparticles were grafted with hydrophilic, biocompatible polyacrylic acid (PAA) with a molecular weight (Mw) of ~1800 amu [32,33]. PAA achieves stable binding to the nanoparticle surface through hard acid (Ln^3+^ of the MMLO nanoparticle)–base (carboxyl groups of PAA) bonding [34,35], forming a robust grafting that prevents aggregation and enables excellent colloidal stability in aqueous solutions. Additionally, these nanoparticles were grafted with small amounts of the organic photosensitizer 2,6-pyridinedicarboxylic acid (PDA) to achieve high fluorescent quantum yields (QYs) with extended fluorescent lifetimes (τs) [36,37,38]. This approach can enhance the versatility and potential utility of MMLO nanoparticles for various biomedical applications, including MRI and FI.

## 2. Results

### 2.1. Particle Diameter, Hydrodynamic Diameter, Colloidal Stability, and Crystallinity of the PAA/PDA-MMLO Nanoparticles

**Figure 1:**
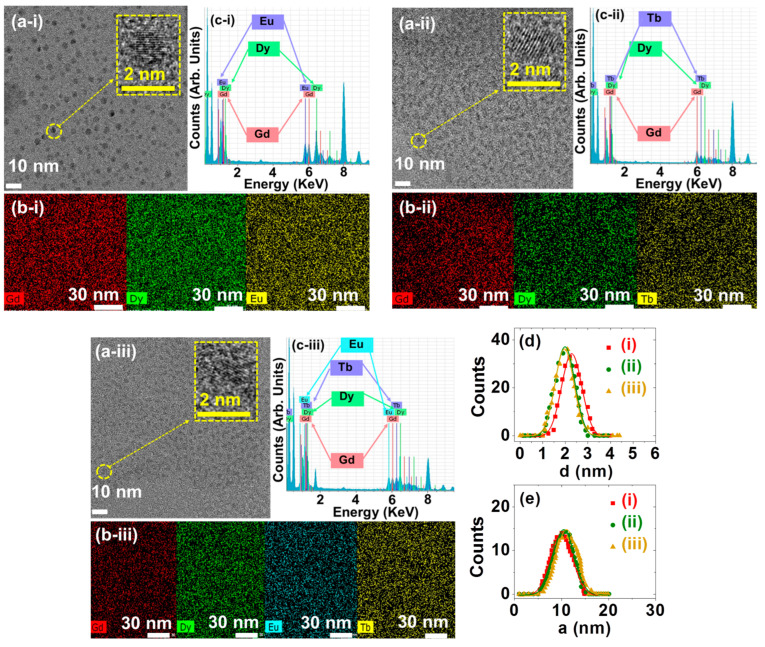
(**a**) HRTEM images, (**b**) elemental mappings of Gd, Dy, Eu, and/or Tb on the HAADF-STEM images, and (**c**) EDS spectra. (**d**) Particle diameter (**d**) distributions and log-normal function fits to obtain d_avg_. (**e**) Hydrodynamic diameter (**a**) distributions and log-normal function fits to obtain a_avg_. Labels (**i**–**iii**) in (**a**–**e**) indicate the PAA/DPA-GDEO, PAA/DPA-GDTO, and PAA/DPA-GDETO nanoparticles, respectively.

The Gd:Dy:Eu:Tb mole ratios in the nanoparticles were determined through inductively coupled plasma-atomic emission spectrometry (ICP-AES). As provided in Table 2, the Dy, Eu, and Tb mole ratios with respect to Gd were nearly 1 for all samples, consistent with the precursor mole ratios used in the synthesis. The high-resolution transmission electron microscope (HRTEM) images revealed that the PAA/PDA-MMLO nanoparticles exhibited ultrasmall particle-size distributions (Figure 1a). Additionally, elemental mappings on the high-angle annular dark-field scanning transmission electron microscopy (HAADF-STEM) images confirmed the uniform elemental distributions of Gd, Dy, Eu, and/or Tb in the nanoparticles (Figure 1b), verifying the uniform mixed nature of various Ln^3+^ in each MMLO nanoparticle. The presence of various Ln^3+^ in the MMLO nanoparticles was demonstrated through energy-dispersive X-ray spectroscopy (EDS) (Figure 1c). The particle-size distributions were fitted to a log-normal function, yielding average particle diameters (d_avg_) of 2.3, 2.0, and 2.0 nm for the PAA/PDA-GDEO, PAA/PDA-GDTO, and PAA/PDA-GDETO nanoparticles, respectively (Figure 1d and Table 2). Moreover, the fitting of the log-normal function to the observed hydrodynamic diameter distributions revealed mean hydrodynamic diameters (a_avg_) of 10.1, 10.5, and 11.1 nm for PAA/PDA-GDEO, PAA/PDA-GDTO, and PAA/PDA-GDETO nanoparticles, respectively (Figure 1e and Table 2). The higher a_avg_ than d_avg_ is attributed to the hydrophilic -COOH groups of PAA, which attracted water molecules near the nanoparticles. The nearly monodispersed particle and hydrodynamic diameters for the PAA/PDA-MMLO nanoparticle samples were confirmed from polydispersity indices (PDI) of <0.1, as listed in Table 2. These findings confirm the successful synthesis of PAA-MMLO and PAA/PDA-MMLO nanoparticles.

**Figure 2:**
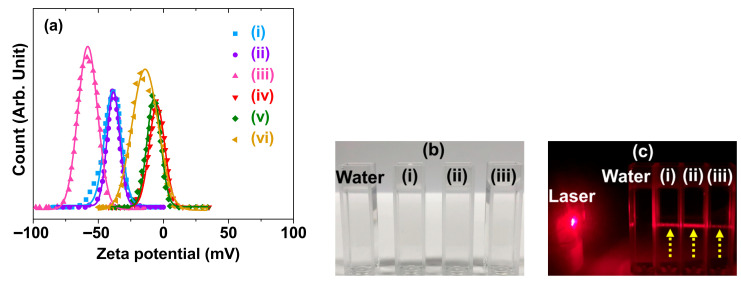
(**a**) Zeta potential (ζ) curves of the aqueous nanoparticle suspension samples and Gaussian function fits to obtain ζ_avg_. (**b**) Photographs of the aqueous nanoparticle suspension samples, exhibiting transparency and no nanoparticle precipitation after synthesis (>1.5 years). (**c**) Laser scattering (indicated with dotted arrows) due to laser collision with nanoparticle colloids in aqueous media, whereas no laser scattering was observed for the vial containing triple-distilled water. The labels (**i**), (**ii**), (**iii**), (**iv**), (**v**), and (**vi**) denote the PAA-GDEO, PAA-GDTO, PAA-GDETO, PAA/PDA-GDEO, PAA/PDA-GDTO, and PAA/PDA-GDETO nanoparticles, respectively.

The zeta potentials (ξ_avg_) were measured to investigate the colloidal stability in aqueous media. The PAA-GDEO, PAA-GDTO, and PAA-GDETO nanoparticles exhibited high zeta potentials of −38.8, −38.3, and −58.1 mV, respectively, confirming their good colloidal stability (Figure 2a and Table 2). These high negative zeta potentials are attributed to the abundant hydrophilic –COOH groups of PAA. However, upon PDA conjugation, the negative zeta potentials decreased to −4.9, −7.2, and −14.0 mV for PAA/PDA-GDEO, PAA/PDA-GDTO, and PAA/PDA-GDETO nanoparticles, respectively (Figure 2a and Table 2), owing to PDA exhibiting lower water solubility than PAA. PDA contains one N and two COO^−^ groups, which enable stronger bonding with Ln^3+^ on the nanoparticle surface than the COO^−^ groups of PAA [39,40]. Importantly, all PAA-MMLO and PAA/PDA-MMLO nanoparticle colloidal samples remained transparent in aqueous media without precipitation for >1.5 years (Figure 2b). As depicted in Figure 2c, the colloidal dispersion was confirmed through laser scattering, also known as the Tyndall effect, which stems from interactions between nanoparticle colloids and the laser, providing evidence that the nanoparticles exist as colloids in aqueous solutions. In contrast, triple-distilled water did not exhibit laser scattering. These findings confirm the good colloidal stability of the nanoparticles in aqueous media, which renders them useful for biomedical applications.

**Figure 3:**
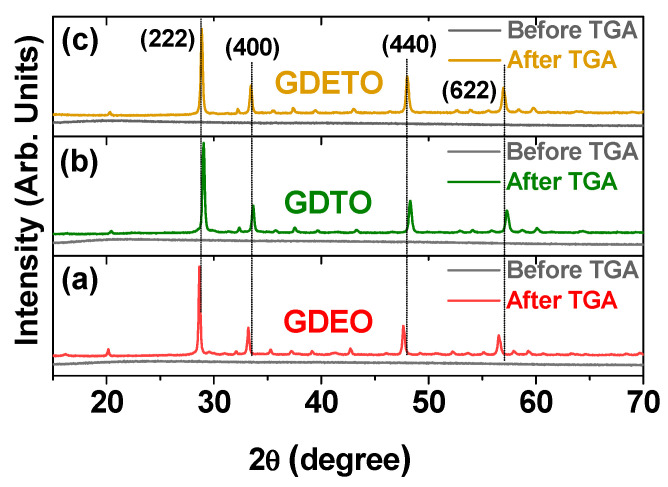
XRD patterns of the powder: (**a**) PAA/PDA-GDEO, (**b**) PAA/PDA-GDTO, and (**c**) PAA/PDA-GDETO nanoparticles before and after TGA up to 900 °C under airflow.

The X-ray diffraction (XRD) patterns of the powder PAA/PDA-GDEO, PAA/PDA-GDTO, and PAA/PDA-GDETO nanoparticles were obtained before and after thermogravimetric analysis (TGA), as depicted in Figure 3a–c, respectively. The initial XRD patterns of the powder samples exhibited amorphous characteristics, indicating incomplete crystallization caused by ultrasmall particle sizes [41]. TGA up to 900 °C under airflow resulted in crystallinity, characterized by sharp peaks. The cubic phase was observed for all samples after TGA. The lattice constants (L) were determined as 10.751, 10.659, and 10.749 Å for the GDEO, GDTO, and GDETO nanoparticles, respectively. These values are consistent with the mole percentage-averaged cell constants of 10.781, 10.731, and 10.762 Å obtained for the GDEO, GDTO, and GDETO nanoparticles, respectively, using single-phase lattice constants of 10.81 Å (Gd_2_O_3_) [42], 10.65 Å (Dy_2_O_3_) [43], 10.84 Å (Eu_2_O_3_) [44], and 10.71 Å (Tb_2_O_3_) [43].

### 2.2. Surface-Grafting of PAA and PDA

**Figure 4:**
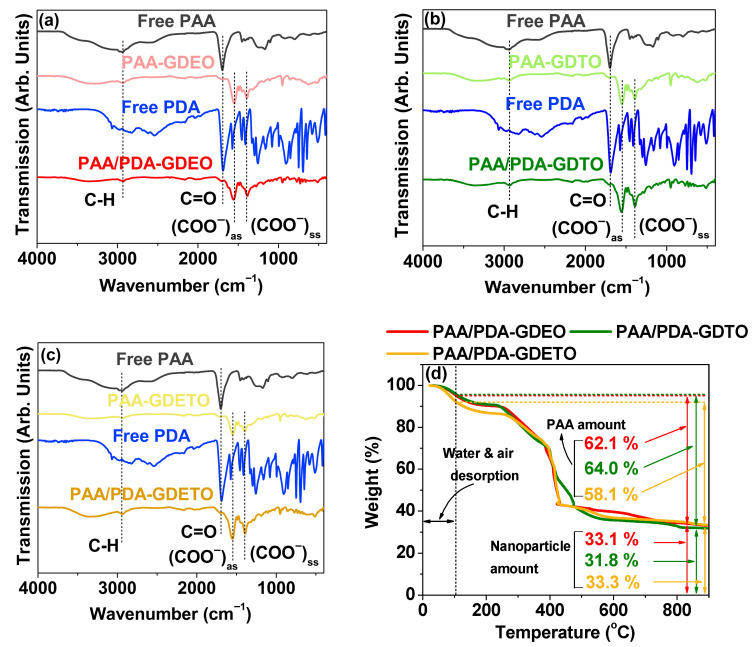
FT-IR absorption spectra of the (**a**) PAA/PDA-GDEO, (**b**) PAA/PDA-GDTO, and (**c**) PAA/PDA-GDETO nanoparticles: “as” and “ss” indicate asymmetric and symmetric COO^−^ stretching vibrations. (**d**) TGA curves of the PAA/PDA-GDEO, PAA/PDA-GDTO, and PAA/PDA-GDETO nanoparticles.

The grafting of PAA and PDA onto the nanoparticle surface was confirmed from the Fourier transform infrared (FT-IR) absorption spectra. The FT-IR absorption spectra of free PAA and PDA were also collected as a reference. As displayed in Figure 4a–c, the C=O stretching vibrations of free PAA at 1698 cm^−1^ and free PDA at 1688 cm^−1^ split and redshifted into antisymmetric and symmetric COO^−^ stretching vibrations at ~1550 and ~1390 cm^−1^, respectively, in the FT-IR absorption spectra of PAA-MMLO and PAA/PDA-MMLO nanoparticles (MMLO = GDEO, GDTO, and GDETO). These results confirm that PAA and PDA were successfully grafted onto nanoparticle surface through hard acid (COO^−^ of PAA and COO^−^ and N of PDA)–base (Ln^3+^ on nanoparticle surface) bonding [34,35,45]. Furthermore, the C–H stretching frequencies of PAA and PDA at ~2935 cm^−1^ in the FT-IR absorption spectra of the PAA-MMLO and PAA/PDA-MMLO nanoparticles also support the successful conjugation of PAA and PDA onto the MMLO nanoparticles. All observed characteristic absorption frequencies are listed in Table 3.

A quantitative evaluation of the PAA/PDA grafting amount (P) on the nanoparticle surface in weight percent (wt. %) was performed using TGA (Figure 4d and Table 2). The evaluation accounted for the initial mass drops that occurred between room temperature and ~105 °C owing to the desorption of water and air from the powder samples. Subsequent mass drops were attributed to the removal of PAA and PDA from the nanoparticles during heating to 900 °C. The remaining masses (Q) were those of neat nanoparticles without coating (Table 2). The grafting densities (σ), which represent the average number of PAA (Mw = 1800 amu) and PDA (Mw = 167.1 amu) molecules grafted per unit surface area of the nanoparticles [46,47], were estimated to be 1.7 nm^−2^ for the PAA/PDA-GDEO nanoparticles, 1.6 nm^−2^ for the PAA/PDA-GDTO nanoparticles, and 1.4 nm^−2^ for the PAA/PDA-GDETO nanoparticles. Since the Mw of PAA is ~11 times higher than that of PDA and the amount of PAA used in the synthesis was ~10 times higher than that of PDA, the above-estimated σ values are likely mostly those of PAA. These estimations were obtained using the P and Q values from the TGA data, d_avg_ values from HRTEM imaging, and mole percentage-weighted bulk densities of 7.546, 7.709, and 7.637 gcm^−3^ for GDEO, GDTO, and GDETO, respectively, based on the bulk densities of 7.407 gcm^−3^ for Gd_2_O_3_, 7.810 gcm^−3^ for Dy_2_O_3_, 7.420 gcm^−3^ for Eu_2_O_3_, and 7.910 gcm^−3^ for Tb_2_O_3_ [48]. The average number of PAA molecules (N_NP_) grafted per nanoparticle surface was estimated to be 28, 20, and 17 for the PAA/PDA-GDEO, PAA/PDA-GDTO, and PAA/PDA-GDETO nanoparticles, respectively, based on the product of σ and πd_avg_^2^. Under these grafting conditions, excellent colloidal stability was observed in aqueous media.

### 2.3. Fluorescent Properties

#### 2.3.1. Optimal PDA Amount and PL Spectra

**Figure 5:**
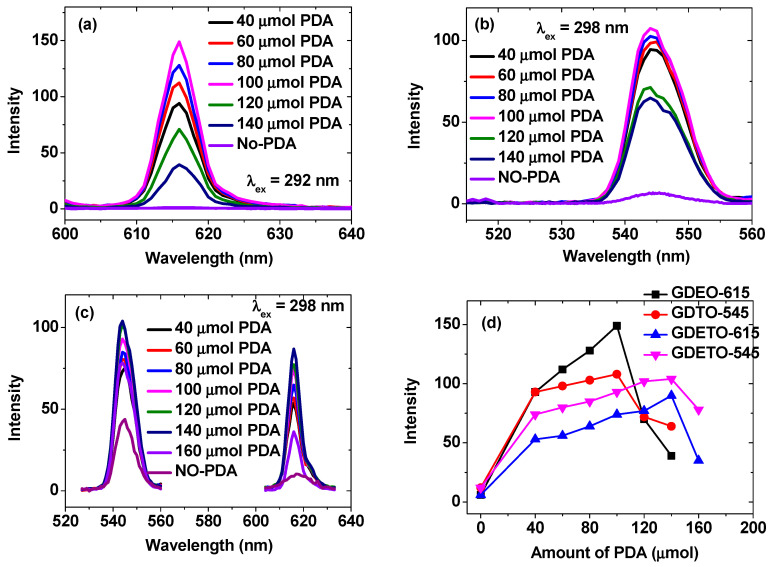
PL spectra of the (**a**) PAA/PDA-GDEO, (**b**) PAA/PDA-GDTO, and (**c**) PAA/PDA-GDETO nanoparticle suspension samples and (**d**) plots of 615-nm and 545-nm peak intensities as functions of the PDA amount added to the nanoparticle suspension samples in aqueous media.

The introduction of a small amount of the PDA into the PAA-MMLO (MMLO = GDEO, GDTO, and GDETO) nanoparticles had a considerable impact on the emission intensities, as shown in Figure 5a–c. This impact was due to the transfer of absorbed energy from PDA to Eu^3+^, Dy^3+^, and/or Tb^3+^ in the MMLO nanoparticles. By measuring the emission intensities at 615 and/or 545 nm and plotting them as a function of the amount of PDA (40–160 μmol) added to 4 mL of the aqueous PAA-MMLO nanoparticle suspension samples (Figure 5d), the optimal amount of PDA was determined to be 100, 100, and 140 μmol PDA for the PAA/PDA-GDEO, PAA/PDA-GDTO, and PAA/PDA-GDETO nanoparticles, respectively. These optimized PAA/PDA-MMLO nanoparticles were subsequently used for various characterizations in this study.

**Figure 6:**
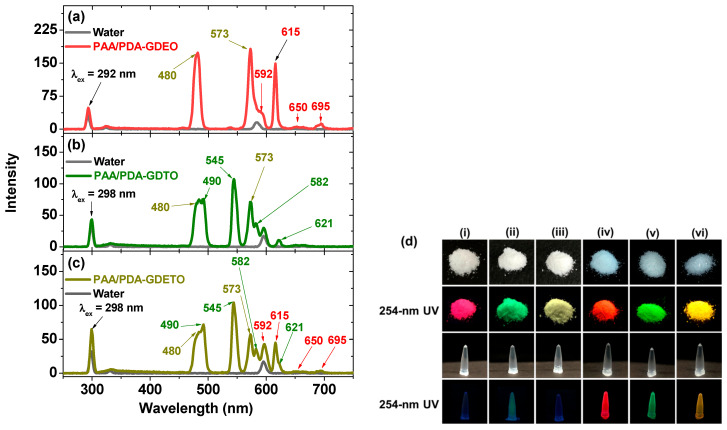
Concentration-normalized PL spectra of the (**a**) PAA/PDA–GDEO, (**b**) PAA/PDA-GDTO, and (**c**) PAA/PDA-GDETO nanoparticle suspension samples in aqueous media. The PL spectra of water are provided for reference. (**d**) Photographs of the powder and aqueous nanoparticle suspension samples with and without 254-nm UV irradiation for (**i**) PAA-GDEO, (**ii**) PAA-GDTO, (**iii**) PAA-GDETO, (**iv**) PDA/PDA-GDEO, (**v**) PDA/PDA-GDTO, and (**vi**) PDA/PDA-GDETO nanoparticles.

As illustrated in Figure 6a–c, the PL spectra of the aqueous suspension samples displayed strong emission peaks at wavelengths of 592 (^5^D_0_→^7^F_1_), 615 (^5^D_0_→^7^F_2_), 650 (^5^D_0_→^7^F_3_), and 695 nm (^5^D_0_→^7^F_4_) from Eu^3+^, 490 (^5^D_4_→^7^F_6_), 545 (^5^D_4_→^7^F_5_), 582 (^5^D_4_→^7^F_4_), and 621 nm (^5^D_4_→^7^F_3_) from Tb^3+^, and 480 (^4^F_9/2_→^6^H_15/2_) and 573 nm (^4^F_9/2_→^6^H_13/2_) from Dy^3+^ in the PAA/PDA-MMLO nanoparticles. Excitation wavelengths (λ_ex_) of 292 and 298 nm were determined from the strongest peaks in the excitation spectra corresponding to the strongest emission wavelengths (λ_em_) of Eu^3+^ at 615 nm and Tb^3+^ at 545 nm in the PL spectra (Table 4). All aqueous suspension and powder samples appeared transparent and white, respectively (Figure 6d). However, under 254-nm ultraviolet (UV) irradiation, the PAA-GDEO, PAA-GDTO, and PAA-GDETO nanoparticles were red, green, and pale-yellow, respectively, while the PAA/PDA-GDEO, PAA/PDA-GDTO, and PAA/PDA-GDETO nanoparticles displayed similar but stronger colors, demonstrating the important role of PDA in enhancing PL [12,49,50].

#### 2.3.2. QY and τ

**Figure 7:**
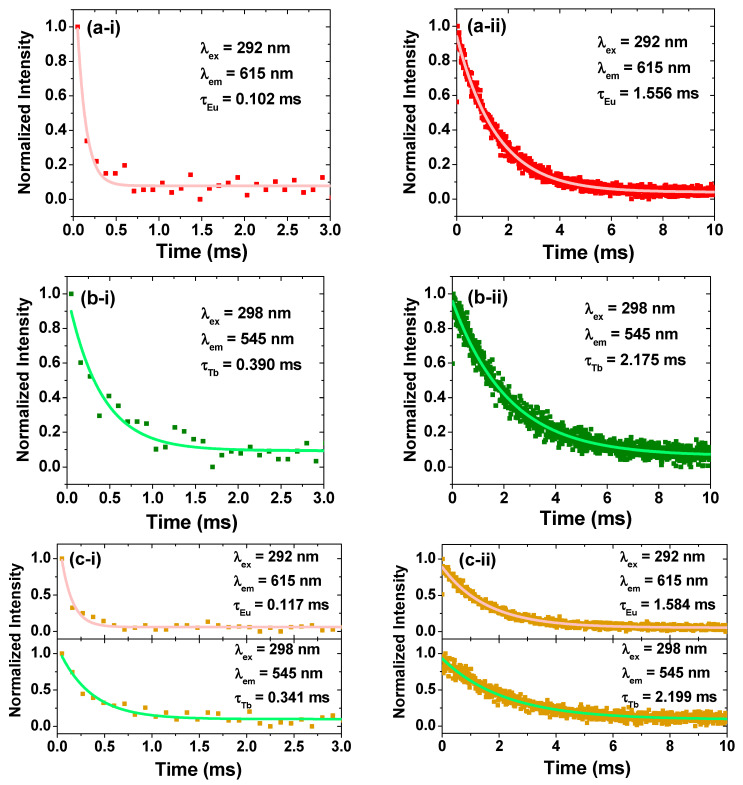
TRF spectra of (**a-i**) PAA-GDEO, (**a-ii**) PAA/PDA-GDEO, (**b-i**) PAA-GDTO, (**b-ii**) PAA/PDA-GDTO, (**c-i**) PAA-GDETO, and (**c-ii**) PAA/PDA-GDETO nanoparticle suspension samples in aqueous media. The intensity was normalized with respect to the initial (i.e., starting) intensity.

The QY and τ are important photophysical parameters for applications, particularly biomedical applications. The absolute QY values were measured using an integration sphere, and the τ values were estimated by recording time-resolved fluorescence (TRF) spectra following exponential decay function fitting, as displayed in Figure 7. As listed in Table 4, the PAA-MMLO (MMLO = GDEO, GDTO, and GDETO) nanoparticles had QY and τ values of 1–2% and 0.1–0.4 ms, respectively, but the PAA/PDA-MMLO nanoparticles exhibited significantly higher QY and τ values of 29–61% and 1.6–2.2 ms, respectively, demonstrating the significant role of PDA in improving the photophysical properties of the nanoparticles via energy transfer. High QY values are essential for the sensitive detection of target molecules or cells, whereas long τ values allow for noise-free detection through time-delayed detection because the τ values of background biological molecules are short (i.e., 0.1–10 ns) [51].

### 2.4. Longitudinal (r_1_) and Transverse (r_2_) Water Proton Spin Relaxivities

**Figure 8:**
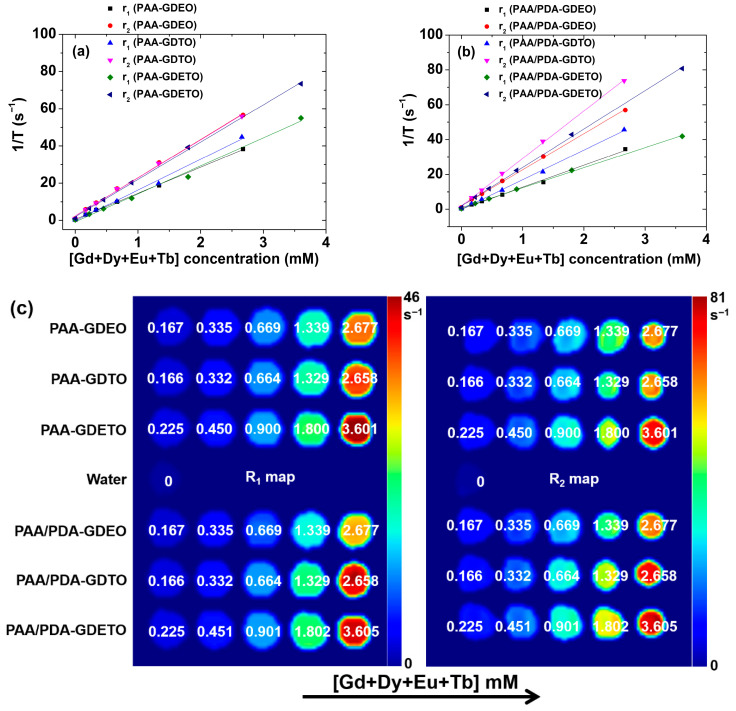
Plots of 1/T_1_ and 1/T_2_ versus Gd + Dy + Eu + Tb concentration in aqueous media for (**a**) PAA-GDEO, PAA-GDTO, and PAA-GDETO nanoparticles and (**b**) PAA/PDA-GDEO, PAA/PDA-GDTO, and PAA/PDA-GDETO nanoparticles. (**c**) R_1_ and R_2_ map images as a function of the Gd + Dy + Eu + Tb concentration, displaying clear dose-dependent contrast enhancements. The numbers in map images are Gd + Dy + Eu + Tb concentrations in mM.

The r_1_ and r_2_ water proton spin relaxivity values were assessed from the linear slopes of the concentration-dependent inverse longitudinal (T_1_) and transverse (T_2_) water proton spin relaxation time plots shown in Figure 8a,b. As listed in Table 5, the PAA-MMLO (MMLO = GDEO, GDTO, and GDETO) nanoparticles had r_1_ and r_2_ values of 14.0–16.6 and 20.1–20.5 s^−1^mM^−1^, respectively, while the PAA/PDA-MMLO nanoparticles had r_1_ and r_2_ values of 11.5–17.0 and 20.7–27.2 s^−1^mM^−1^, respectively. The high r_1_ and r_2_ values are reflected by strong dose-dependent contrast changes in the longitudinal (R_1_) and transverse (R_2_) map images, as shown in Figure 8c, demonstrating in vitro that the PAA-MMLO and PAA/PDA-MMLO nanoparticles can function as T_1_ and/or T_2_ MRI contrast agents.

## 3. Discussion

**Figure 9:**
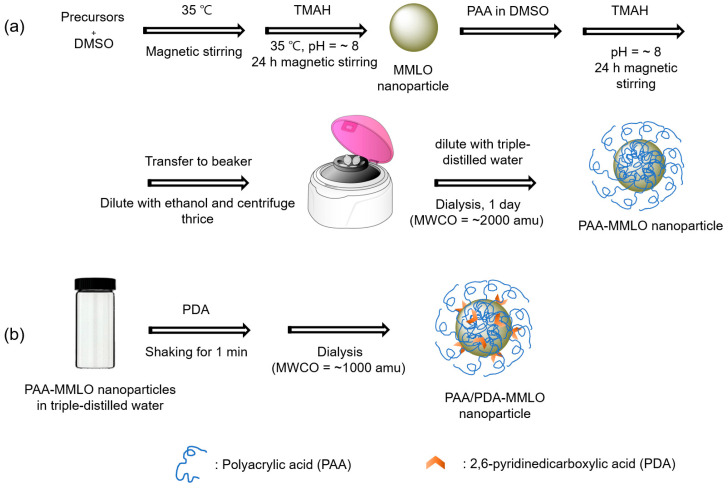
Synthesis of (**a**) PAA-MMLO nanoparticles using a one-pot method. (**b**) Conjugation of PDA with the PAA-MMLO nanoparticles to obtain PAA/PDA-MMLO nanoparticles. In (**a**,**b**), MMLO = GDEO, GDTO, and GDETO.

PAA-MMLO (MMLO = GDEO, GDTO), and GDETO) nanoparticles were successfully synthesized using a one-pot method (Figure 9a). Subsequently, they were conjugated with a small amount of the PDA to yield PAA/PDA-MMLO nanoparticles (Figure 9b). The HRTEM images revealed that all nanoparticles were ultrasmall with average particle diameters of ~2 nm, and their colloidal stability in aqueous media was excellent without precipitation after synthesis (>1 year), making them useful for biomedical applications. The excellent colloidal stability was attributed to PAA grafting on the nanoparticle surfaces.

The PAA-MMLO and PAA/PDA-MMLO nanoparticles exhibited high r_1_ and r_2_ values, which were 4–5 and 6–7 times higher than those of commercial molecular contrast agents [17,18], respectively. These findings demonstrate that the PAA-MMLO and PAA/PDA-MMLO nanoparticles can function as T_1_ and/or T_2_ MRI contrast agents. The r_1_ and r_2_ values are comparable to those of Gd-containing nanoparticles such as PAA-Gd_2_O_3_ [52], glycosamine-PAA-Gd_2_O_3_ [52], PAAMA-Gd_2_O_3_ [53], carbon-Gd_2_O_3_ [42], D-gluconic acid-GDO [30], and lactobionic acid-Gd_0.74_Eu_1.26_O_3_ nanoparticles [55], but considerably higher than those of Gd-free nanoparticles such as PAA-Dy_2_O_3_ [54], carbon-Dy_2_O_3_ [20], and PAA-Tb_2_O_3_ nanoparticles [24], as presented in Table 5. This is due to the different 4f-electron configurations; Gd^3+^ (^8^S_7/2_) is more efficient in inducing T_1_ and T_2_ water proton spin relaxations than Dy^3+^ (^6^H_15/2_) and Tb^3+^ (^7^F_6_) [18]. The nanoparticles possess high surface-to-volume (S/V) ratios owing to their ultrasmall particle diameters of ~2 nm, enabling them to have enhanced water proton spin relaxivities. The r_1_ and r_2_ values are affected by particle size [56,57]. The r_1_ value is optimal at ultrasmall particle diameters of 1.1–2.5 nm, whereas r_2_ value increases with increasing particle size [57]. This is because the r_1_ value is mostly affected by the surface Gd^3+^ and the S/V ratio is the highest at ultrasmall particle size, whereas the r_2_ value is influenced by the nanoparticle magnetic moment, which increases with particle size. For cytotoxicity, hydrophilic, biocompatible ligand-grafted lanthanide oxide nanoparticles are nontoxic up to high Ln-concentration [58]. For example, carboxyfluorescein–polyethylene glycol-grafted gadolinium oxide nanoparticles were nontoxic up to 8 mM [Gd] [58]. In addition, polymaleic acid-grafted ultrasmall gadolinium oxide nanoparticles (d = 2.1 nm) exhibited predominant renal excretion owing to their ultrasmall particle size and thus, minimal body accumulation in mice [59]; this will considerably lower the potential risk of in vivo toxicity. Moreover, Gd_2_O_3_:Eu^3+^ nanoparticles (d = 7.4 nm) in BALB/c mice exhibited minimal immunotoxicity [60]. These previous biosafety results suggest that the PAA-MMLO and PAA/PDA-MMLO nanoparticles will be nontoxic and thus useful for in vivo biomedical applications.

The PAA/PDA-MMLO nanoparticles showed significantly improved QY and τ values owing to photosensitizing effects of PDA. The strong PL intensity of the PAA/PDA-MMLO nanoparticles in the visible region holds significant promise for various applications as FI agents. Furthermore, the high QY values and long τ values indicate that the PAA/PDA-MMLO nanoparticles can be used for highly sensitive and noise-free detection.

The advantage of MMLO nanoparticles compared to unmixed Ln_2_O_3_ nanoparticles includes optical property tunability, such as frequency and color, and multimodality as imaging agents in MRI and FI. The strong PL intensities of the PAA/PDA-MMLO nanoparticles in aqueous media at 480 nm (blue), 573 nm (yellow), and 615 nm (red) for MMLO = GDEO, 480 nm (blue), 490 nm (blue), 545 nm (green), 573 nm (yellow), 582 nm (yellow) for MMLO = GDTO, and 480 nm (blue), 490 nm (blue), 545 nm (green), 573 nm (yellow), and 615 nm (red) for MMLO = GDETO (Figure 6a), demonstrate their frequency and color tunability. Therefore, high r_1_ and r_2_ values (r_2_/r_1_ = ~1.5) as well as strong PL intensities demonstrate their potential as multimodal imaging agents in T_1_ and/or T_2_ MRI and FI.

## 4. Materials and Methods

### 4.1. Chemicals

GdCl_3_·*x*H_2_O (99.9%), DyCl_3_·*x*H_2_O (99.9%), EuCl_3_·*x*H_2_O (99.99%), TbCl_3_·*x*H_2_O (99.99%), NaOH (99.99%), PAA (Mw = ~1800 amu), dimethyl sulfoxide (DMSO, 99.9%), and trimethylammonium hydroxide pentahydrate (TMAH; ≥97%) were purchased from Sigma-Aldrich (St. Louis, MO, USA) and used as received. PDA (99%) was purchased from Tokyo Chemical Industry (Tokyo, Japan) and used as received. Ethanol (EtOH; 99%, Duksan, Ansan, Republic of Korea) was used for the initial washing of the nanoparticles. Triple-distilled water was used for the final washing of the nanoparticles and the preparation of nanoparticle colloidal solutions.

### 4.2. Synthesis

#### 4.2.1. Synthesis of PAA-MMLO Nanoparticles

Ultrasmall MMLO (i.e., GDEO, GDTO, and GDETO) nanoparticles grafted with hydrophilic, biocompatible PAA were synthesized using a one-pot method (Figure 9a). Briefly, in a three-necked round-bottom flask suspended on a silicon oil bath on a hot plate with temperature and magnetic stirring control, 1 mmol of each Ln-precursor (Ln = Gd, Dy, Eu, and/or Tb) was dissolved together in 25 mL of DMSO through magnetic stirring to obtain a clear solution. Next, 2.9 mL of TMAH was added, and the mixture was magnetically stirred at 35 °C for 24 h to yield a white cloudy mixture. Subsequently, 1 mmol of PAA was added for surface grafting of the MMLO nanoparticles. The pH of the solution was maintained at ~8 by adding TMAH, and the solution was stirred for an additional 24 h at 35 °C. The resulting product solution was washed three times with 400 mL of EtOH through centrifugation. Thereafter, it was dialyzed against 1 L of triple-distilled water using a dialysis tube (molecular weight cut off, MWCO = ~2000 amu) with magnetic stirring for one day to remove any remaining impurities; the triple-distilled water was replaced thrice during dialysis. One-half of the sample was dried to a powder form for various characterizations, and the remaining half was dispersed in triple-distilled water to prepare an aqueous colloidal suspension.

#### 4.2.2. Synthesis of PAA/PDA-MMLO Nanoparticles

The PAA/PDA-MMLO nanoparticles were prepared by simply shaking the PAA-MMLO nanoparticle suspension sample after adding PDA to it (Figure 9b). PDA can strongly bind to the MMLO nanoparticle through its two –COO^−^ groups and one N group. The optimal amount of PDA was determined by adding small amounts of PDA (40–140 μmol) to 4 mL of PAA-MMLO nanoparticle colloidal suspension, followed by shaking for one min to obtain PAA/PDA-MMLO nanoparticles. The mixture was dialyzed against 1 L of triple-distilled water (MWCO = ~1000 amu) to remove free PDA, and then the PL intensity was measured as a function of PDA amount. The optimal PDA amount was determined based on the sample that yielded the highest PL intensity.

### 4.3. Analysis of the PAA/PDA-MMLO Nanoparticles

The diameters of the PAA/PDA-MMLO nanoparticles were measured using an HRTEM (Titan G2 ChemiSTEM CS Probe, FEI, Hillsboro, OR, USA) operating at 200 kV. For the measurements, one drop of aqueous nanoparticle suspension was applied onto a carbon film supported by a 200-mesh copper grid (PELCO no. 160, Ted Pella, Inc., Redding, CA, USA) placed on filter paper using a micropipette (2–20 mL, Eppendorf, Hamburg, Germany). The copper grid containing the sample was air-dried at room temperature before being loaded into the HRTEM for measurements. The crystal structures of the powder samples before and after TGA were characterized using a multi-purpose powder X-ray diffractometer (X-PERT PRO MRD, Philips, Eindhoven, The Netherlands) with unfiltered CuKa (λ = 1.54184 Ǻ) radiation. The scanning step and scan range in 2θ were set to 0.03° and 15–100°, respectively. The surface grafting of PAA and PAA/PDA onto the MMLO nanoparticles was investigated by recording their FT-IR absorption spectra (Galaxy 7020A, Mattson Instruments, Inc., Madison, WI, USA) in the range of 400–4000 cm^−1^. To this end, the powder samples were dried on a hot plate at 40 °C for 1 week to remove moisture. The pellets of the dried powder samples were prepared in KBr. To estimate the surface-grafting amount of PAA/PDA on the MMLO nanoparticles, the TGA curves (SDT-Q600, TA Instruments, New Castle, DE, USA) were recorded between room temperature and 900 °C under airflow. The surface-grafting amount was estimated from the mass drops in the TGA curves after subtracting the initial mass drops that occurred between room temperature and ~105 °C owing to water and air desorption. The Ln concentration in aqueous suspensions of the PAA-MMLO and PAA/PDA-MMLO nanoparticles (Ln = Gd, Dy, Eu, and/or Tb) was measured through ICP-AES (Avio500, Perkin Elmer, Waltham, MA, USA).

### 4.4. Acquisition of the PL Spectra, Absolute Fluorescent QYs, and τs

A PL spectrometer (Cary Eclipse, Agilent Technologies, Santa Clara, CA, USA) was used to acquire the PL spectra of aqueous suspensions of the PAA-MMLO and PAA/PDA-MMLO nanoparticles. A quartz cuvette with four optically clear sides (3 mL, Sigma-Aldrich) was filled with a suspension sample to collect the PL spectra. The absolute QY values were measured using an integrating sphere installed in a PL spectrometer (Hitachi, F-7000, Tokyo, Japan) and a 0.2-mL polypropylene tube filled with the colloidal sample, with another polypropylene tube serving as a reference. The τ values were measured by collecting the TRF spectra and fitting them with an exponential decay function.

### 4.5. Measurements of Water Proton Spin Relaxivities and Map Images

A 3.0 T MRI scanner (Magnetom Trio Tim, Siemens, Munich, Bayern, Germany) was used to measure the T_1_ and T_2_ water proton spin relaxation times and obtain the R_1_ and R_2_ map images at 22 °C. Various aqueous nanoparticle suspension samples (concentrations: 1.0, 0.5, 0.25, 0.125, 0.0625, and 0.0 mM Gd) were prepared by diluting the original concentrated suspension samples with triple-distilled water. These diluted samples were used to measure the T_1_ and T_2_ relaxation times and obtain the R_1_ and R_2_ map images. The T_1_ relaxation times were measured using an inversion recovery method, and the T_2_ relaxation times were measured using the Carr–Purcell–Meiboom–Gill pulse sequence for multiple spin-echo measurements. Next, the r_1_ and r_2_ water proton spin relaxivities were estimated from the slopes of plots of 1/T_1_ and 1/T_2_ versus the Gd + Dy + Eu or Gd + Dy + Tb or Gd + Dy + Eu + Tb concentration, respectively.

## 5. Conclusions

In this study, we used a simple one-pot method to synthesize MMLO nanoparticles comprising three or four lanthanide elements, such as GDEO, GDTO, and GDETO nanoparticles. The MMLO nanoparticles were grafted with hydrophilic, biocompatible PAA for colloidal stability and biocompatibility and a small amount of an organic photosensitizer, PDA, for enhanced QYs and τs, as summarized below.

(1) The PAA-MMLO and PAA/PDA-MMLO nanoparticles were nearly monodispersed and ultrasmall, with an average particle diameter of ~2 nm. They exhibited excellent colloidal stability without precipitation after synthesis for over 1 year.
(2) The PAA-MMLO nanoparticles exhibited r_1_ and r_2_ values of 14.0–16.6 and 20.1–20.5 s^−1^mM^−1^, respectively, whereas the PAA/PDA-MMLO nanoparticles exhibited r_1_ and r_2_ values of 11.5–17.0 and 20.7–27.2 s^−1^mM^−1^, respectively. These r_1_ and r_2_ values are 4–5 and 6–7 times, respectively, higher than those of commercial molecular contrast agents.
(3) The PAA-MMLO nanoparticles had QY and τ values of 1–2% and 0.1–0.4 ms, respectively, whereas the PAA/PDA-MMLO nanoparticles had significantly higher QY and τ values of 29–61% and 1.6–2.2 ms, respectively, underscoring the significant role of PDA in improving the photophysical properties of lanthanides via energy transfer and thus enhancing their potential for biomedical applications.
(4) These findings confirm the potential utility of PAA-MMLO nanoparticles as T_1_ and/or T_2_ MRI contrast agents, as well as PAA/DPA-MMLO nanoparticles as both FI agents and T_1_ and/or T_2_ MRI contrast agents.

## Figures and Tables

**Table 1:** Summarized properties of lanthanide (Ln^3+^) ions used in this study.

Ln^3+^	Ground State Configuration	Fluorescent Color	Application Field	Ref.
Gd^3+^	^8^S_7/2_	Colorless	T_1_ MRI	[17,18,19]
Dy^3+^	^6^H_15/2_	Pale Yellow	T_2_ MRI, FI	[20,21,22]
Eu^3+^	^7^F_0_	Red	FI	[12,25,26]
Tb^3+^	^7^F_6_	Green	T_2_ MRI, FI	[23,24,25,26,27,28,29]

**Table 2:** Summary of the observed physicochemical properties of the PAA/PDA-GDEO, PAA/PDA-GDTO, and PAA/PDA-GDETO nanoparticles.

Sample	Gd:Dy:Eu:Tb Mole Ratio from ICP-AES	d_avg_ (nm)	PDI	a_avg_ (nm)	PDI	ξ_avg_ (mV)		Surface-Grafting Result		
						Without PDA	With PDA	Q (P) (wt. %)	σ (nm^−2^)	N_NP_
GDEO	1.0(Gd):0.92(Dy):0.96(Eu)	2.3	0.05	10.1	0.06	−38.8	−4.9	33.1 (62.1)	1.7	27.6
GDTO	1.0(Gd):0.93(Dy):0.93(Tb)	2.0	0.07	10.5	0.06	−38.3	−7.2	31.8 (64.0)	1.6	19.9
GDETO	1.0(Gd):0.94(Dy):0.93(Eu):0.93(Tb)	2.0	0.07	11.1	0.05	−58.1	−14.0	33.3 (58.9)	1.4	17.3

**Table 3:** Observed FT-IR absorption frequencies in cm^−1^.

Nanoparticle	C–H Stretch	C=O Stretch	COO^−^ Antisymmetric Stretch	COO^−^ Symmetric Stretch
PAA	2936	1698	-	-
PDA	-	1688	-	-
PAA-GDEO	2935	1710	1549	1392
PAA-GDTO		1708	1549	1389
PAA-GDETO		1709	1547	1394
PAA/PDA-GDEO	2935	1706	1556	1389
PAA/PDA-GDTO	2935	1706	1554	1388
PAA/PDA-GDETO	2936	1701	1554	1390

**Table 4:** Summary of the photophysical properties of the PAA-MMLO and PAA/PDA-MMLO nanoparticles in aqueous media.

Sample	Fluorescent Color	λ_ex_ (nm)	λ_em_ (nm)	τ (ms)	QY (%)
PAA-GDEO	Pale red	292	615	0.102	1
PAA-GDTO	Pale green	298	545	0.390	1
PAA-GDETO	Pale yellow	298	615	0.117	2
			545	0.341	
PAA/PDA-GDEO	Strong red	292	615	1.556	45
PAA/PDA-GDTO	Strong green	298	545	2.175	29
PAA/PDA-GDETO	Strong yellow	298	615	1.589	61
			545	2.199	

**Table 5:** Water proton spin relaxivities (r_1_ and r_2_ values).

Nanoparticle (Ln Used in the Plot as Concentration)	Ligand	r_1_ (s^−1^ mM^−1^)	r_2_ (s^−1^ mM^−1^)	r_2_/r_1_	Applied Field (T)	Ref.
GDEO (Gd + Dy + Eu)	PAA	14.03	20.54	1.46	3.0	This study
	PAA + PDA	12.59	20.70	1.64		
GDTO (Gd + Dy + Tb)	PAA	16.55	20.46	1.24	3.0	This study
	PAA + PDA	16.98	27.23	1.60		
GDET (Gd + Dy + Eu + Tb)	PAA	15.04	20.06	1.33	3.0	This study
	PAA + PDA	11.51	22.06	1.92		
Gd_2_O_3_ (Gd)	PAA	14.31	24.09	1.68	1.5	[52]
	PAA + GlcN	10.18	18.38	1.80		
	PAA	19.32	34.23	1.77	3.0	
	PAA + GlcN	12.60	24.46	1.92		
Gd_2_O_3_ (Gd)	PAAMA	40.60	63.40	1.56	3.0	[53]
Gd_2_O_3_ (Gd)	Carbon	16.26	24.12	1.48	1.5	[42]
Tb_2_O_3_ (Tb)	PAA	0.10	3.19	31.90	3.0	[24]
		0.30	16.40	54.67	9.4	
Dy_2_O_3_ (Dy)	PAA	0.16	2.01	12.56	3.0	[54]
		0.55	11.31	20.56	9.4	
Dy_2_O_3_ (Dy)	Carbon	0.10	5.70	57.00	3.0	[20]
GDO (Gd)	D-glucuronic acid	12.60	83.60	6.60	1.5	[30]
GDO (Dy)		11.60	76.80	6.60		
GDO (Gd + Dy)		6.00	40.00	6.70		
Gd_0.74_Eu_1.26_O_3_	Lactobionic acid	11.90	38.70	3.25	1.5	[55]

## Data Availability

The original contributions presented in this study are included in the article. Further inquiries can be directed to the corresponding authors.
